# Osteopathic Approach in the Stimulation of the Endocannabinoid System: A Case Report

**DOI:** 10.7759/cureus.54848

**Published:** 2024-02-24

**Authors:** Caterina Buonomini, Oliviero Bonetti, Emanuele Novelli

**Affiliations:** 1 Osteopathic, Biological, Medical Research, Omicron Beta Research, Roma, ITA; 2 Facoltà di Medicina e Chirurgia, Università di Roma Tor Vergata, Roma, ITA

**Keywords:** compression of fourth ventricle, endocannabinoid system, autonomic nervous system modulation, cranial ostheopathy, osteopathic manipulative medicine

## Abstract

The endocannabinoid system is an important neuromodulatory system responsible for maintaining homeostasis in the biological system. Similarly, the autonomic nervous system is important to control involuntary bodily functions such as thermoregulation, regulation of heart rate and sleep latency. The endocannabinoid system and the autonomic nervous system can be modified by osteopathic manipulative techniques. In this report, we present a case of a 53-year-old patient with comorbidities, habitual cannabis use, and disorders associated with the endocannabinoid system and autonomic nervous system, on whom the cranial technique of the fourth ventricle (CV4) was performed. The results obtained from the Pittsburgh Questionnaire and the Health Survey 36 (SF-36) Questionnaire were encouraging. The patient decreased tetrahydrocannabinol (THC) consumption. Her sleep quality and health improved, thus improving her quality of life. CV4 aimed to influence the functioning of the patient's autonomic nervous system and consequently the endocannabinoid system. Further research using randomized controlled trials will be important to have measurable and objective data on this topic.

## Introduction

The endocannabinoid system (ECS) is primarily responsible for maintaining homeostasis, balancing the internal environment and energy input and output in living biological systems, as well as regulating physiological processes. CB1 and CB2 cannabinoid receptors are the primary targets of endogenous cannabinoids (endocannabinoids). These G protein-coupled receptors play an important role in many processes, including metabolic regulation, craving, pain, anxiety, bone growth, and immune function [1]. Previously published studies have drawn connections between the ECS, autonomic nervous system (ANS), and cranial osteopathic techniques [2,3]. The ECS and ANS are two distinct but interconnected systems present in the human body. They influence each other and promote regulation of the dopaminergic system. The fourth ventricle compression (CV4) is a well-known osteopathic procedure, first described by Sutherland in 1939 [4]. CV4 is a cranial manipulation technique aimed at influencing the functioning of the autonomic nervous system [5]. This technique is also used to reduce anxiety and pain, decrease sleep latency and sympathetic activity. We decided to carry out this approach because it is now known that osteopathic treatment can increase the release of cannabinoid receptors (CB), but we wanted to understand if this would be possible using only the CV4 technique [6]. Here, we report a clinical case where it is thought that cranial osteopathic technique stimulated the endocannabinoid system through the autonomic nervous system. 

## Case presentation

**Figure 1:**
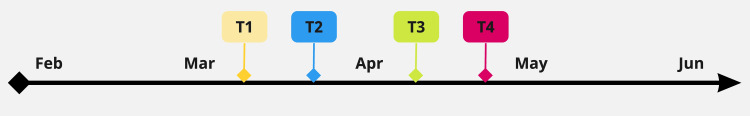
Timeline of intervention from March 2023 to May 2023

**Figure 2:**
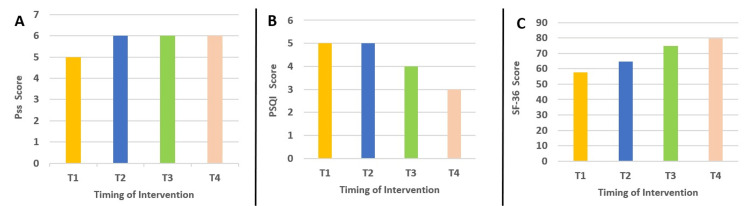
Histograms of total score results of (A) Perceived Stress Scale (PSS), (B) Pittsburgh Sleep Quality Index (PSQI); and (C) Health Survey 36 (SF-36) health questionnaire obtained by osteopathic manipulative treatment protocol (A) Slight improvement in the results obtained from the PSS; (B) clear improvement in the results of the PSQI; (C) progressive improvement in the SF-36 health questionnaire

The patient is a 53-year-old female who has been using cannabinoids for 25 years. She takes phytocannabinoids to sleep, lower her stress and reduce chronic pain. She had thyroid cancer which was removed in 2006. She underwent three radioiodine ablations between 2006 and 2010. She is following pharmacological therapy composed of L-thyroxine (synthetic) 125mcg for six days a week and half a tablet of L-thyroxine (synthetic) 125mcg one day a week. She also takes Livial® 2.5mcg as a sexual hormone therapy replacement. In July 2023 she contracted Lyme disease, which created many problems with her lymphatic system, heaviness, tiredness, fever, and underarm lymph node reaction. She was treated with doxycycline 100mg for 57 consecutive days. The patient tested negative in November 2023. The disease left sequelae on her as weakness, severe insomnia and swollen underarm lymph nodes until the second osteopathic treatment. During this period the patient usually consumed 5-6g of cannabinoids (THC) every month. We waited for five months after her results were negative before starting the CV4 technique. The case report was developed from March 2023 until May 2023. During this period the patient was treated twice a month (Figure 1). CARE report guidelines were used to write the case report [7]. The osteopath who carried out the protocol has more than 10 years of experience in osteopathy and more than 25 years in physiotherapy. The duration of treatment was approximately 20 minutes each time and sessions were conducted at the same time and in the same medical room to try to keep the patient as comfortable as possible. Before starting the accurate study of this case report, the osteopath performed a 30-minute training session to estimate the reference points, the pressure applied, and possible problems that might arise while performing the treatment. The patient was subjected to the Health Survey 36 (SF-36) questionnaires, the Pittsburgh Questionnaire and the Perceived Stress Scale (PSS) [8-10] at the beginning of the case, however more objective measures are needed in future research. Subsequently, she was subjected to the questionnaires before and after each scheduled treatment (four treatments). The total score of the three questionnaires was calculated at the end of each treatment session. The CV4 has been used according to the osteopathic manipulative protocol. The technique was performed as follows. The procedure of CV4 appears to be simple; the patient lies down, and the therapist holds the squamous part of the occipital bone, specifically its lateral angles, while manipulating the cranium into an extension. The therapist maintains the extension of the cranium and waits for a motionless state. Once the cranial rhythmic impulse (CRI) becomes apparent, the therapist may end the procedure [11]. The CRI physiological phenomenon is associated with ANS activity. The results obtained in the different questionnaires were (Figure 2): regarding the PSS at time t1=5; t2=6; t3=6; t4=6. For the Pittsburgh Sleep Quality Index (PSQI) at t1=5; t2=5; t3=4; t4=3. For the SF-36 Health Questionnaire t1=57.7; t2=64.86; t3=74.86; t4=77.91. Almost completely unchanged were the total scores for the PSS. There was a slight improvement in the PSQI and an improvement in the SF-36; however, more objective measures are needed.

## Discussion

The endocannabinoid system plays a very important role in the survival of the human body, and endocannabinoids are substances that our body naturally produces under the stimulation of receptors in this system such as CB1 and CB2. This is due to their ability to play a critical role in maintaining the homeostasis of the human body - brain, endocrine and immune systems included, to name a few [12]. Endocannabinoids play a therapeutic role in the treatment of pain, neurodegenerative diseases and inflammatory conditions, including spasticity and pain in multiple sclerosis [13]. The endocannabinoid system, similarly to the autonomic nervous system, is distributed in a complex anatomical network. The endocannabinoid system and autonomic nervous systems are interconnected, and even though they have different receptors, they travel in the brainstem with the diffuse neuromodulatory systems. By diffuse neuromodulatory systems, we mean systems that originate in the brainstem and have extensive connections with different areas of the central nervous system with regulatory functions, such as attention, learning, memory, mood, analgesia, behavior and drug addiction. Examples are the noradrenergic system (NA) which originates at the level of the locus coeruleus, the serotoninergic system (5-HT) which originates at the level of the raphe nuclei, the dopaminergic system (DA) which originates from the substantia nigra and from the tegmental area, and the cholinergic system (Ach) which originates from the basal forebrain complex, from the medial septal nuclei and the nucleus of Meynert. The autonomic nervous system is critical for the proper functioning of vital human systems such as thermoregulation, regulation of heart rate and blood pressure, and other fundamental physiological processes [14]. Recent research has shown how the use of cannabidiol (CBD) can have a mimetic effect on the parasympathetic system by acting on vagal afferents. In addition, it has been shown to be effective in conditions such as epilepsy, depression, and anxiety that to date are treatable by vagus nerve stimulation [15]. However, it is important to emphasize that the relationship between ECS and ANS is still being studied and should be understood more deeply. Much research is needed to fully explore this interaction and its effects on human health. Cranial manipulation such as the CV4 affects sympathetic and parasympathetic nervous system-mediated physiological parameters such as blood flow velocity, sleep latency, and stress modulation [16,17]. More specifically, it is thought that it may decrease the activity of the sympathetic system and increase the activity of the parasympathetic system. The results obtained from the scores of each questionnaire were interesting. After the osteopathic protocol program, the patient exceeded the minimum detectable change score for all parameters taken into consideration. In particular, there were significant improvements in the SF-36 and Pittsburg questionnaires. No interesting results were obtained from the perceived stress questionnaire. The patient decreased her THC consumption from 5-6 g/month to 1 g/month. After two months, all improved values were maintained over time.

## Conclusions

We believe that through the CV4 technique we were able to influence the ECS. In the case of this patient, CV4 stimulation brought significant results. Through this case report, we confirmed the benefits that osteopathic techniques can have on patients with chronic pain and the help they could give in decreasing cannabis use in people who use it. It is encouraging for us to note how the results of questionnaires showed that some basic functions of the autonomic nervous system such as sleep and quality of life improved. This case report needs laboratory instruments and new techniques to achieve its goals on a larger scale. We recommend further research involving more patients and using randomized controlled trials with more effective tools and validated measures.
